# Supplementary material for: The impact of agricultural industrial agglomeration on farmers’ income: An influence mechanism test based on a spatial panel model
Source: PLoS One. 2023 Sep 8;18(9):e0291188. doi: 10.1371/journal.pone.0291188 (PMC10491000; doi:10.1371/journal.pone.0291188)
Supplement: S4 Appendix — (DOCX) [file pone.0291188.s004.docx]

**1 The** **impact of agro-industrial agglomeration on farmers' income-the spatial panel model**

Moran test

| 年份 | **The economic-geography nested weight** | | | **The geographical weight** | | |
| --- | --- | --- | --- | --- | --- | --- |
|  | **Moran’ I** | **Z** | **P** | **Moran’ I** | **Z** | **P** |
| 2019 | 0.094 | 3.407 | 0.001 | 0.086 | 3.450 | 0.001 |
| 2018 | 0.094 | 3.382 | 0.001 | 0.085 | 3.370 | 0.001 |
| 2017 | 0.089 | 3.242 | 0.001 | 0.080 | 3.206 | 0.001 |
| 2016 | 0.097 | 3.459 | 0.001 | 0.084 | 3.352 | 0.001 |
| 2015 | 0.094 | 3.357 | 0.001 | 0.079 | 3.203 | 0.001 |
| 2014 | 0.091 | 3.300 | 0.001 | 0.076 | 3.119 | 0.002 |
| 2013 | 0.090 | 3.277 | 0.001 | 0.074 | 3.050 | 0.002 |

1.1 LM test：Whether to use the spatial panel model

| Test | Statistic | df | p-value |
| --- | --- | --- | --- |
| Spatial error: | | | |
| Moran's I | 3.880 | 1 | 0.000 |
| Lagrange multiplier | 10.892 | 1 | 0.001 |
| Robust Lagrange multiplier | 1.264 | 1 | 0.261 |
| Spatial lag: | | | |
| Lagrange multiplier | 88.348 | 1 | 0.000 |
| Robust Lagrange multiplier | 78.719 | 1 | 0.000 |

1.2 Hausman test：fixed effect

chi2(6) = (b-B)'[(V_b-V_B)^(-1)](b-B) = 13.25

Prob > chi2 = 0.0392

1.3 LR test：two-way fixed effect

| lrtest both ind,df(10) | |
| --- | --- |
| Likelihood-ratio test | LR chi2(10) = 79.92 |
| Assumption: ind nested in both | Prob > chi2 = 0.0000 |
| lrtest both time,df(10) | |
| Likelihood-ratio test | LR chi2(10) = 463.26 |
| Assumption: time nested in both | Prob > chi2 = 0.0000 |

1.4 WALD test：the results are the same as the LR test

| (1) [Wx]gather - [Wx]GDP = 0 | (1) [Wx]gather = -[Spatial]rho*[Main]gather |
| --- | --- |
| (2) [Wx]gather - [Wx]credit = 0 | (2) [Wx]GDP = -[Spatial]rho*[Main]GDP |
| (3) [Wx]gather - [Wx]edu = 0 | (3) [Wx]credit = -[Spatial]rho*[Main]credit |
| (4) [Wx]gather - [Wx]invest = 0 | (4) [Wx]edu = -[Spatial]rho*[Main]edu |
| (5) [Wx]gather - [Wx]machine = 0 | (5) [Wx]invest = -[Spatial]rho*[Main]invest |
| (6) [Wx]gather = 0 | (6) [Wx]machine = -[Spatial]rho*[Main]machine |
| chi2(6) = 65.05 | chi2(6) = 43.70 |
| Prob > chi2 = 0.0000 | Prob > chi2 = 0.0000 |

1.5 LR test：SDM cannot degenerate to SEM and SAR, so SDM with two-way fixed effects is chosen

| lrtest sdm sar | |
| --- | --- |
| Likelihood-ratio test | LR chi2(6) = 49.44 |
| Assumption: sar nested within sdm | Prob > chi2 = 0.0000 |
| lrtest sdm sem | |
| Likelihood-ratio test | LR chi2(6) = 38.40 |
| Assumption: sem nested within sdm | Prob > chi2 = 0.0000 |

1.6 Regression results of the impact of agro-industry agglomeration on farmers' income

|  | **(1)Ordinary panel model** | **(2)SDM** | **(3)SEM** | **(4)SLR** |
| --- | --- | --- | --- | --- |
|  | **income** | **income** | **income** | **income** |
| main |  |  |  |  |
| gather | 0.255** | 0.048*** | 0.035** | 0.035** |
|  | (0.101) | (0.014) | (0.014) | (0.014) |
| GDP | 0.450*** | 0.128*** | 0.123*** | 0.121*** |
|  | (0.127) | (0.023) | (0.019) | (0.020) |
| credit | 0.275*** | 0.023** | 0.031*** | 0.031*** |
|  | (0.046) | (0.010) | (0.010) | (0.010) |
| edu | 0.060* | 0.008 | 0.001 | 0.001 |
|  | (0.030) | (0.009) | (0.009) | (0.009) |
| invest | -0.135** | -0.019* | -0.009 | -0.009 |
|  | (0.052) | (0.010) | (0.010) | (0.010) |
| machine | -0.035 | 0.029** | 0.018 | 0.018 |
|  | (0.051) | (0.012) | (0.012) | (0.012) |
| _cons | 2.472** |  |  |  |
|  | (1.031) |  |  |  |
| Wx |  |  |  |  |
| gather |  | 0.212** |  |  |
|  |  | (0.099) |  |  |
| GDP |  | 0.076 |  |  |
|  |  | (0.113) |  |  |
| credit |  | 0.268*** |  |  |
|  |  | (0.065) |  |  |
| edu |  | 0.072 |  |  |
|  |  | (0.047) |  |  |
| invest |  | -0.067 |  |  |
|  |  | (0.067) |  |  |
| machine |  | -0.099 |  |  |
|  |  | (0.073) |  |  |
| Spatial |  |  |  |  |
| rho |  | -0.104 |  | 0.107 |
|  |  | (0.242) |  | (0.208) |
| lambda |  |  | 0.017 |  |
|  |  |  | (0.208) |  |
| Variance |  |  |  |  |
| sigma2_e |  | 0.000*** | 0.000*** | 0.000*** |
|  |  | (0.000) | (0.000) | (0.000) |
| N | 210.000 | 210.000 | 210.000 | 210.000 |
| r2 | 0.910 | 0.360 | 0.455 | 0.476 |
| AIC | -644.305 | -1100.555 | -1099.688 | -1100.014 |
| Log-likelihood | 328.1525 | 564.2773 | 557.8438 | 558.0071 |

1.8 Direct, indirect and total effects of each variable

| **variables** | **direct effects** | | **indirect effects** | | **total effects** | |
| --- | --- | --- | --- | --- | --- | --- |
|  | **SDM** | **SLR** | **SDM** | **SLR** | **SDM** | **SLR** |
| gather | 0.047*** | 0.035** | 0.194** | 0.006 | 0.241*** | 0.041** |
|  | (0.014) | (0.014) | (0.085) | (0.011) | (0.085) | (0.018) |
| GDP3 | 0.126*** | 0.120*** | 0.052 | 0.023 | 0.178** | 0.142*** |
|  | (0.020) | (0.018) | (0.085) | (0.038) | (0.083) | (0.040) |
| credit | 0.022** | 0.032*** | 0.252*** | 0.005 | 0.274*** | 0.037** |
|  | (0.010) | (0.010) | (0.064) | (0.011) | (0.064) | (0.015) |
| edu | 0.008 | 0.002 | 0.071 | 0.000 | 0.079 | 0.002 |
|  | (0.010) | (0.010) | (0.054) | (0.003) | (0.059) | (0.012) |
| invest2 | -0.021* | -0.011 | -0.061 | -0.003 | -0.081 | -0.013 |
|  | (0.012) | (0.011) | (0.066) | (0.007) | (0.071) | (0.016) |
| machine2 | 0.030*** | 0.019* | -0.090 | 0.004 | -0.060 | 0.023 |
|  | (0.011) | (0.011) | (0.067) | (0.007) | (0.066) | (0.014) |

**2 The impact of agro-industrial agglomeration on farmers` income structure**

**Regression results of spatial panel model**

|  | **LM test** | **Rho/lambda** | **Direct and inderect effect** |
| --- | --- | --- | --- |
| Proportion of wage income | significant | Non-significant | Non-significant |
| Proportion of operation income | Non-significant | - | - |
| Proportion of property income | significant | Non-significant | Non-significant |
| Proportion of transfer income | significant | significant | Non-significant |

Therefore, the ordinary panel model are used for this part.

**2.1** **The impact of agro-industrial agglomeration on the proportion of farmers' wage income**

2.1.1 Hausman test：the statistic is negative.

chi2(6) = (b-B)'[(V_b-V_B)^(-1)](b-B) = -30.30 chi2 < 0

2.1.2 an over-identification test: fixed effect

Sargan-Hansen statistic 214.020 Chi-sq(6) P-value = 0.0000

2.1.3 LSDV: Most of the individual dummy variables are significant, and the null hypothesis "all individual dummy variables are 0" is rejected, that is, there is an individual effect.

| 1.pro | 0.000 | 7.pro | -0.114** | 13.pro | -0.507*** | 19.pro | -0.149 | 25.pro | -0.421*** |
| --- | --- | --- | --- | --- | --- | --- | --- | --- | --- |
|  | (.) |  | (0.045) |  | (0.089) |  | (0.092) |  | (0.069) |
| 2.pro | -0.530*** | 8.pro | -0.359*** | 14.pro | -0.549*** | 20.pro | -0.366*** | 26.pro | -0.371*** |
|  | (0.076) |  | (0.071) |  | (0.086) |  | (0.076) |  | (0.080) |
| 3.pro | -0.588*** | 9.pro | -0.435*** | 15.pro | -0.303*** | 21.pro | -0.469*** | 27.pro | -0.437*** |
|  | (0.081) |  | (0.091) |  | (0.084) |  | (0.085) |  | (0.057) |
| 4.pro | 0.125** | 10.pro | -0.395*** | 16.pro | -0.363*** | 22.pro | -0.366*** | 28.pro | -0.344*** |
|  | (0.061) |  | (0.100) |  | (0.082) |  | (0.099) |  | (0.086) |
| 5.pro | -0.559*** | 11.pro | -0.213** | 17.pro | -0.278*** | 23.pro | -0.533*** | 29.pro | -0.465*** |
|  | (0.076) |  | (0.090) |  | (0.095) |  | (0.070) |  | (0.057) |
| 6.pro | -0.483*** | 12.pro | -0.248*** | 18.pro | -0.442*** | 24.pro | -0.325*** | 30.pro | -0.608*** |
|  | (0.078) |  | (0.074) |  | (0.097) |  | (0.070) |  | (0.088) |

2.1.4 F test: there is no time fixed effect.

F(6,29) = 1.44 prob > F = 0.2348

According to the above results, the individual fixed effects model is selected.

**2.2 The impact of agro-industrial agglomeration on the proportion of farmers' operation income**

2.2.1 Hausman test: fixed effect

chi2(6) = (b-B)'[(V_b-V_B)^(-1)](b-B) = 115.50

Prob > chi2 = 0.0000

2.2.2 LSDV: Most of the individual dummy variables are significant, and the null hypothesis "all individual dummy variables are 0" is rejected, that is, there is an individual effect.

| 1.pro | 0.000 | 7.pro | 0.168*** | 13.pro | 0.458*** | 19.pro | 0.200*** | 25.pro | 0.393*** |
| --- | --- | --- | --- | --- | --- | --- | --- | --- | --- |
|  | (.) |  | (0.031) |  | (0.070) |  | (0.070) |  | (0.062) |
| 2.pro | 0.551*** | 8.pro | 0.341*** | 14.pro | 0.576*** | 20.pro | 0.470*** | 26.pro | 0.418*** |
|  | (0.065) |  | (0.070) |  | (0.072) |  | (0.107) |  | (0.054) |
| 3.pro | 0.556*** | 9.pro | 0.376*** | 15.pro | 0.305*** | 21.pro | 0.422*** | 27.pro | 0.324*** |
|  | (0.055) |  | (0.061) |  | (0.055) |  | (0.058) |  | (0.043) |
| 4.pro | -0.082 | 10.pro | 0.423*** | 16.pro | 0.359*** | 22.pro | 0.332*** | 28.pro | 0.286*** |
|  | (0.059) |  | (0.066) |  | (0.059) |  | (0.063) |  | (0.058) |
| 5.pro | 0.595*** | 11.pro | 0.182*** | 17.pro | 0.340*** | 23.pro | 0.460*** | 29.pro | 0.293*** |
|  | (0.053) |  | (0.063) |  | (0.062) |  | (0.058) |  | (0.070) |
| 6.pro | 0.402*** | 12.pro | 0.207*** | 18.pro | 0.387*** | 24.pro | 0.360*** | 30.pro | 0.623*** |
|  | (0.058) |  | (0.048) |  | (0.064) |  | (0.056) |  | (0.074) |

2.2.3 F test: there is a time effect.

F(6,29) = 5.94 prob > F = 0.0004

According to the above results, the double fixed effects model is selected.

**2.3 The impact of agro-industrial** **agglomeration on the proportion of farmers' property income**

2.3.1 Hausman test：Random effect

chi2(7) = (b-B)'[(V_b-V_B)^(-1)](b-B) = 8.29 Prob>chi2 =0.3077

2.3.5 LM test: The individual effect mode

Chibar2(01) = 445.65 Prob>chibar2 = 0.0000

According to the above results, the individual random effect model is selected.

**2.4 The impact of agro-industrial agglomeration on the proportion of farmers' transfer income**

2.4.1 Hausman test: fixed effect

chi2(7) = (b-B)'[(V_b-V_B)^(-1)](b-B) = 19.82

Prob > chi2 = 0.0060

2.4.2 LSDV: Most of the individual dummy variables are insignificant, and the null hypothesis "all individual dummy variables are 0" is accepted, that is, there is no individual effect.

| 1.pro | 0.000 | 7.pro | -0.067 | 13.pro | 0.067 | 19.pro | -0.040 | 25.pro | 0.059 |
| --- | --- | --- | --- | --- | --- | --- | --- | --- | --- |
|  | (.) |  | (0.044) |  | (0.097) |  | (0.096) |  | (0.085) |
| 2.pro | -0.003 | 8.pro | 0.038 | 14.pro | -0.012 | 20.pro | -0.086 | 26.pro | -0.032 |
|  | (0.089) |  | (0.078) |  | (0.094) |  | (0.103) |  | (0.081) |
| 3.pro | 0.026 | 9.pro | 0.075 | 15.pro | -0.007 | 21.pro | 0.065 | 27.pro | 0.125** |
|  | (0.085) |  | (0.097) |  | (0.086) |  | (0.089) |  | (0.063) |
| 4.pro | -0.075 | 10.pro | -0.026 | 16.pro | 0.024 | 22.pro | 0.048 | 28.pro | 0.075 |
|  | (0.055) |  | (0.103) |  | (0.087) |  | (0.101) |  | (0.087) |
| 5.pro | -0.025 | 11.pro | 0.046 | 17.pro | -0.056 | 23.pro | 0.097 | 29.pro | 0.176*** |
|  | (0.081) |  | (0.094) |  | (0.100) |  | (0.084) |  | (0.063) |
| 6.pro | 0.092 | 12.pro | 0.042 | 18.pro | 0.065 | 24.pro | -0.012 | 30.pro | -0.032 |
|  | (0.087) |  | (0.076) |  | (0.102) |  | (0.073) |  | (0.105) |

2.4.3 F test: time effect

F(6,29) = 4.47 prob > F = 0.0025

According to the above results, the time fixed effect model is selected.

**2.5 Regression results**

|  | **(1)the individual fixed effects mode** | **(2)the two-way fixed effects mode** | **(3)The individual random effect mode** | **(4)the time fixed effects model** |
| --- | --- | --- | --- | --- |
|  | **Proportion of wage income** | **Proportion of operation income** | **Proportion of property income** | **Proportion of transfer income** |
| main |  |  |  |  |
| gather | 0.036^**^ | -0.025^*^ | 0.001 | -0.021^***^ |
|  | (0.017) | (0.013) | (0.002) | (0.005) |
| GDP | 0.024 | 0.039^**^ | 0.006^**^ | -0.083^***^ |
|  | (0.022) | (0.019) | (0.003) | (0.010) |
| credit | 0.016^*^ | 0.028^***^ | 0.001 | -0.008 |
|  | (0.009) | (0.009) | (0.001) | (0.007) |
| edu | -0.032^**^ | 0.012 | 0.001 | -0.022^***^ |
|  | (0.015) | (0.009) | (0.002) | (0.007) |
| invest | -0.023 | 0.013 | 0.002 | 0.019^***^ |
|  | (0.020) | (0.009) | (0.002) | (0.007) |
| machine | 0.020 | -0.036^***^ | -0.004^***^ | 0.002 |
|  | (0.016) | (0.011) | (0.001) | (0.003) |
| _cons | 0.211 | -0.127 | -0.043 | 1.453^***^ |
|  | (0.322) | (0.202) | （0.028） | (0.141) |
| N | 210.000 | 210.000 | 210.000 | 210.000 |
| r2 | 0.4650 | 0.3421 | 0.3176 | 0.3817 |

**3 The impact of agro-industrial agglomeration on farmers` wage, operation, property and transfer income**

**Regression results of spatial panel model**

|  | **LM test** | **Rho/lambda** | **Direct effect** | **Inderect effect** |
| --- | --- | --- | --- | --- |
| wage income | significant | Non-significant | Non-significant | significant |
| operation income | Non-significant | - | - |  |
| property income | significant | Non-significant | Non-significant | Non-significant |
| transfer income | significant | Non-significant | significant | Non-significant |

Therefore, the ordinary panel model are used for this part.

**3.1 The impact of agro-industrial agglomeration on farmers` wage income**

Hausman test：fixed effect

chi2(6) = (b-B)'[(V_b-V_B)^(-1)](b-B) = 102.02

Prob>chi2 = 0.0000

**3.2** **The impact of agro-industrial agglomeration on farmers` operation income**

Hausman test：fixed effect

chi2(7) = (b-B)'[(V_b-V_B)^(-1)](b-B) = 39.17

Prob>chi2 = 0.0000

**3.3 The impact of agro-industrial agglomeration on farmers` property income**

Hausman test：fixed effect

chi2(6) = (b-B)'[(V_b-V_B)^(-1)](b-B) = 23.78

Prob>chi2 = 0.0006

**3.4 The impact of agro-industrial agglomeration on farmers` transfer income**

Hausman test：the statistic is negative.

chi2(6) = (b-B)'[(V_b-V_B)^(-1)](b-B) = -8.58 chi2 < 0

an over-identification test: fixed effect

Sargan-Hansen statistic 30.822 Chi-sq(6) P-value = 0.0000

**3.5 Regression results**

|  | **Wage income** | **Operating income** | **Property income** | **Transfer income** |
| --- | --- | --- | --- | --- |
| gather | 0.378^***^ | 0.149^***^ | 0.348^***^ | 0.550^***^ |
|  | (0.062) | (0.042) | (0.113) | (0.101) |
| GDP | 0.528^***^ | 0.398^***^ | 0.570^***^ | 0.521^***^ |
|  | (0.086) | (0.058) | (0.157) | (0.139) |
| credit | 0.329^***^ | 0.186^***^ | 0.379^***^ | 0.343^***^ |
|  | (0.037) | (0.025) | (0.068) | (0.059) |
| edu | 0.008 | 0.007 | 0.101 | 0.210^***^ |
|  | (0.043) | (0.029) | (0.080) | (0.070) |
| invest | -0.212^***^ | -0.024^*^ | -0.067 | -0.313^***^ |
|  | (0.046) | (0.031) | (0.084) | (0.073) |
| machine | 0.066 | -0.019 | -0.082 | -0.105 |
|  | (0.054) | (0.037) | (0.010) | (0.088) |
| _cons | 0.211 | 2.419^***^ | -4.182^***^ | -0.312 |
|  | (0.827) | (0.562) | (1.516) | (1.344) |
| N | 210.000 | 210.000 | 210.000 | 210.000 |
| r2 | 0.8518 | 0.8297 | 0.6973 | 0.7504 |
| F | 81.52*** | 90.24*** | 34.87*** | 23.38*** |

3.6 To compare the differences in variable coefficients among the four models, the coefficient difference test based on the Seemingly Unrelated Regression Estimation (SUR) was used. However, since STATA does not support such test for panel data, the individual effects in the model were first removed manually, and then the OLS estimation was used. Finally, the coefficient difference test based on the Seemingly Unrelated Regression Estimation (SUR) was used.

There are significant differences between the coefficients of the wage income model and the operation income model

[income1_mean]gather - [income2_mean]gather = 0

chi2( 1) = 80.51 Prob > chi2 = 0.0000

The rest of the tables are omitted.

**4 Mediation effect test**

**4.1 The the mediating effect of agro-industrial agglomeration on farmers' income_the three-step test**

4.1.1 model 1

(1)Hausman test：fixed effect

chi2(6) = (b-B)'[(V_b-V_B)^(-1)](b-B) = 52.08

Prob>chi2 = 0.0000

(2)LSDV: Most of the individual dummy variables are significant, and the null hypothesis "all individual dummy variables are 0" is rejected, that is, there is an individual effect.

| 1.pro | 0.000 | 7.pro | -0.063 | 13.pro | -0.497** | 19.pro | -0.101 | 25.pro | -0.787*** |
| --- | --- | --- | --- | --- | --- | --- | --- | --- | --- |
|  | (.) |  | (0.082) |  | (0.221) |  | (0.213) |  | (0.222) |
| 2.pro | -0.515** | 8.pro | -0.630*** | 14.pro | -0.628*** | 20.pro | -0.554** | 26.pro | -0.345** |
|  | (0.219) |  | (0.199) |  | (0.210) |  | (0.253) |  | (0.172) |
| 3.pro | -0.683*** | 9.pro | -0.298 | 15.pro | -0.385** | 21.pro | -0.318* | 27.pro | -0.520*** |
|  | (0.191) |  | (0.230) |  | (0.194) |  | (0.192) |  | (0.157) |
| 4.pro | 0.259** | 10.pro | -0.238 | 16.pro | -0.420** | 22.pro | -0.387* | 28.pro | -0.687*** |
|  | (0.123) |  | (0.227) |  | (0.210) |  | (0.222) |  | (0.191) |
| 5.pro | -0.266 | 11.pro | -0.359* | 17.pro | -0.360 | 23.pro | -0.875*** | 29.pro | -0.409** |
|  | (0.175) |  | (0.216) |  | (0.234) |  | (0.240) |  | (0.174) |
| 6.pro | -0.408* | 12.pro | -0.245 | 18.pro | -0.305 | 24.pro | -0.488*** | 30.pro | -0.489** |
|  | (0.214) |  | (0.164) |  | (0.237) |  | (0.171) |  | (0.234) |

(3)F test: Time effect

F(6,29) = 142.8 Prob > F = 0.0000

According to the above results, the two-way fixed effect model is selected.

4.1.2 model 2

(1) Hausman test：fixed effect

chi2(6) = (b-B)'[(V_b-V_B)^(-1)](b-B) =22.97

Prob>chi2 = 0.0008

(2) LSDV: Most of the individual dummy variables are significant, and the null hypothesis "all individual dummy variables are 0" is rejected, that is, there is an individual effect.

| 1.pro | 0.000 | 7.pro | 1.043*** | 13.pro | 2.951*** | 19.pro | 2.835*** | 25.pro | 2.452*** |
| --- | --- | --- | --- | --- | --- | --- | --- | --- | --- |
|  | (.) |  | (0.367) |  | (0.829) |  | (0.868) |  | (0.735) |
| 2.pro | 2.863*** | 8.pro | 1.204* | 14.pro | 1.349* | 20.pro | 0.533 | 26.pro | 2.275*** |
|  | (0.776) |  | (0.707) |  | (0.780) |  | (0.720) |  | (0.721) |
| 3.pro | 2.081*** | 9.pro | 3.218*** | 15.pro | 2.075** | 21.pro | 2.933*** | 27.pro | 2.952*** |
|  | (0.785) |  | (0.906) |  | (0.837) |  | (0.810) |  | (0.588) |
| 4.pro | 1.719*** | 10.pro | 3.372*** | 16.pro | 2.712*** | 22.pro | 2.679*** | 28.pro | 2.551*** |
|  | (0.421) |  | (0.989) |  | (0.768) |  | (0.907) |  | (0.762) |
| 5.pro | 1.829** | 11.pro | 4.079*** | 17.pro | 3.165*** | 23.pro | 1.958** | 29.pro | 1.568*** |
|  | (0.732) |  | (0.786) |  | (0.941) |  | (0.766) |  | (0.573) |
| 6.pro | 2.605*** | 12.pro | 2.511*** | 18.pro | 3.220*** | 24.pro | 2.102*** | 30.pro | 1.801** |
|  | (0.812) |  | (0.700) |  | (0.972) |  | (0.675) |  | (0.871) |

(3) F test: Time effect

F(6,29) = 14.60 Prob > F = 0.0000

According to the above results, the two-way fixed effects model is selected.

4.1.3 model 3

(1) Hausman test：fixed effect

chi2(6) = (b-B)'[(V_b-V_B)^(-1)](b-B) = 28.74

Prob>chi2 = 0.0002

(2) LSDV: Most of the individual dummy variables are significant, and the null hypothesis "all individual dummy variables are 0" is rejected, that is, there is an individual effect.

| 1.pro | 0.000 | 7.pro | -0.175** | 13.pro | -0.813*** | 19.pro | -0.405* | 25.pro | -1.050*** |
| --- | --- | --- | --- | --- | --- | --- | --- | --- | --- |
|  | (.) |  | (0.072) |  | (0.234) |  | (0.217) |  | (0.241) |
| 2.pro | -0.822*** | 8.pro | -0.759*** | 14.pro | -0.773*** | 20.pro | -0.611*** | 26.pro | -0.589*** |
|  | (0.240) |  | (0.188) |  | (0.196) |  | (0.210) |  | (0.181) |
| 3.pro | -0.906*** | 9.pro | -0.643** | 15.pro | -0.608*** | 21.pro | -0.632*** | 27.pro | -0.836*** |
|  | (0.197) |  | (0.264) |  | (0.202) |  | (0.209) |  | (0.190) |
| 4.pro | 0.075 | 10.pro | -0.599** | 16.pro | -0.710*** | 22.pro | -0.674*** | 28.pro | -0.960*** |
|  | (0.076) |  | (0.250) |  | (0.233) |  | (0.241) |  | (0.204) |
| 5.pro | -0.462** | 11.pro | -0.796*** | 17.pro | -0.699*** | 23.pro | -1.085*** | 29.pro | -0.577*** |
|  | (0.182) |  | (0.260) |  | (0.270) |  | (0.262) |  | (0.156) |
| 6.pro | -0.687*** | 12.pro | -0.514*** | 18.pro | -0.650** | 24.pro | -0.713*** | 30.pro | -0.682*** |
|  | (0.239) |  | (0.182) |  | (0.273) |  | (0.170) |  | (0.231) |

(3) F test: Time effect

F(6,29) = 110.41 Prob > F = 0.0000

According to the above results, the two-way fixed effects model is selected.

4.1.4 the three-step test

|  | 1. **The two-way fixed**   **effect model** | 1. **the two-way fixed**   **effect model** | 1. **The two-way fixed**   **effect model** |
| --- | --- | --- | --- |
|  | **income** | **firm** | **income** |
| gather | 0.035^**^ | 0.291^**^ | 0.028^*^ |
|  | (0.015) | (0.134) | (0.015) |
| firm |  |  | 0.025^***^ |
|  |  |  | (0.009) |
| GDP | 0.123^***^ | 0.055 | 0.121^***^ |
|  | (0.021) | (0.189) | (0.021) |
| credit | 0.031^***^ | 0.008 | 0.031^***^ |
|  | (0.011) | (0.093) | (0.010) |
| edu | 0.001 | -0.312^***^ | 0.009 |
|  | (0.010) | (0.087) | (0.010) |
| invest | 0.009 | -0.120^**^ | -0.006 |
|  | (0.011) | (0.095) | (0.011) |
| machine | 0.018 | 0.207^*^ | -0.013 |
|  | (0.013) | (0.113) | (0.013) |
| _cons | 7.450^***^ | 10.804^***^ | 7.175^***^ |
|  | (0.231) | (2.023) | (0.244) |
| N | 210.000 | 210.000 | 210.000 |
| r2 | 0.9899 | 0.7805 | 0.9904 |
| F | 292.60*** | 45.22*** | 300.91*** |

4.1.5 bootstrap

|  | Observed | Bootstrap |  |  | Normal-based | |
| --- | --- | --- | --- | --- | --- | --- |
|  | Coef. | Std.Err. | z | P>\|z\| | 95% Conf. | Interval |
| _bs_1 | -0.0060 | 0.0062 | -0.98 | 0.328 | -0.018 | 0.006 |
| _bs_2 | 0.0952 | 0.0202 | 4.72 | 0.000 | 0.056 | 0.135 |

**4.2 The the mediating effect of agro-industrial agglomeration on farmers' wage income**

4.2.1 model 1

Hausman test：fixed effect

chi2(6) = (b-B)'[(V_b-V_B)^(-1)](b-B) = 102.02

Prob>chi2 = 0.0000

4.2.2 model 2

Hausman test：fixed effect

chi2(6) = (b-B)'[(V_b-V_B)^(-1)](b-B) = 22.97

Prob>chi2 = 0.0008

4.2.3 model 3

Hausman test：fixed effect

chi2(6) = (b-B)'[(V_b-V_B)^(-1)](b-B) = 156.77

Prob>chi2 = 0.0000

4.2.4 the three-step test

|  | **(1)** | **(2)** | **(3)** |
| --- | --- | --- | --- |
|  | **Wage income** | **Agricultural organization** | **Wage income** |
| gather | 0.378*** | 0.801*** | 0.269*** |
|  | (0.062) | (0.158) | (0.062) |
| firm |  |  | 0.136*** |
|  |  |  | (0.028) |
| GDP | 0.528*** | 0.846*** | 0.413*** |
|  | (0.086) | (0.219) | (0.084) |
| credit | 0.329*** | 0.380*** | 0.278*** |
|  | (0.037) | (0.094) | (0.036) |
| edu | 0.008 | -0.230** | 0.039 |
|  | (0.043) | (0.111) | (0.041) |
| invest | -0.212*** | -0.436*** | -0.153*** |
|  | (0.046) | (0.117) | (0.045) |
| machine | 0.066 | -0.126 | 0.083 |
|  | (0.054) | (0.139) | (0.051) |
| _cons | 0.211 | 2.686 | -0.153 |
|  | (0.827) | (2.117) | (0.781) |
| N | 210.000 | 210.000 | 210.000 |
| r2 | 0.852 | 0.605 | 0.870 |
| F | 81.52*** | 31.86*** | 82.28*** |

**4.3 The the mediating effect of agro-industrial agglomeration on farmers' operation income**

4.3.1 model 1

Hausman test：fixed effect

chi2(6) = (b-B)'[(V_b-V_B)^(-1)](b-B) = 39.17

Prob>chi2 = 0.0000

4.3.2 model 2

Hausman test：fixed effect

chi2(6) = (b-B)'[(V_b-V_B)^(-1)](b-B) = 22.97

Prob>chi2 = 0.0008

4.3.3 model 3

Hausman test：fixed effect

chi2(6) = (b-B)'[(V_b-V_B)^(-1)](b-B) = 30.65

Prob>chi2 = 0.0001

4.3.4 the three-step test

|  | **(4)** | **(5)** | **(6)** |
| --- | --- | --- | --- |
|  | **Operating income** | **Agricultural organization** | **Operating income** |
| gather | 0.149*** | 0.801*** | 0.098** |
|  | (0.042) | (0.158) | (0.044) |
| firm |  |  | 0.062*** |
|  |  |  | (0.020) |
| GDP | 0.398*** | 0.846*** | 0.345*** |
|  | (0.058) | (0.219) | (0.059) |
| credit | 0.186*** | 0.380*** | 0.162*** |
|  | (0.025) | (0.094) | (0.026) |
| edu | 0.007 | -0.230** | 0.022 |
|  | (0.030) | (0.111) | (0.029) |
| invest | -0.024 | -0.436*** | 0.004 |
|  | (0.031) | (0.117) | (0.031) |
| machine | -0.019 | -0.126 | -0.011 |
|  | (0.037) | (0.139) | (0.036) |
| _cons | 2.419*** | 2.686 | 2.251*** |
|  | (0.562) | (2.117) | (0.551) |
| N | 210.000 | 210.000 | 210.000 |
| r2 | 0.830 | 0.605 | 0.839 |
| F | 90.24*** | 31.86*** | 91.79*** |

4.4 To compare the differences in variable coefficients among the two models, the coefficient difference test based on the Seemingly Unrelated Regression Estimation (SUR) was used. However, since STATA does not support such test for panel data, the individual effects in the model were first removed manually, and then the OLS estimation was used. Finally, the coefficient difference test based on the Seemingly Unrelated Regression Estimation (SUR) was used.

There are significant differences between the coefficients of the wage income mediation model and the operation income mediation model.

[income1_mean]gather - [income2_mean]gather = 0

chi2( 1) = 79.36 Prob > chi2 = 0.0000
