# Supplementary material for: The impact of agricultural industrial agglomeration on farmers’ income: An influence mechanism test based on a spatial panel model
Source: PLoS One. 2023 Sep 8;18(9):e0291188. doi: 10.1371/journal.pone.0291188 (PMC10491000; doi:10.1371/journal.pone.0291188)
Supplement: Attachment — Submitted filename: Response to Reviewers.doc [file pone.0291188.s006.doc]

Response to reviewers

Dear editor and reviewers,

Thank you for offering us an opportunity to improve the quality of our submitted manuscript(”The impact of agricultural industrial agglomeration on farmers' income: an influence mechanism test based on a spatial panel model ”). We appreciated very much the reviewers’ constructive comments. In this revision, we have addressed all of these comments. We hope the revised manuscript has now met the publication standard of our journal.

We highlighted all the revisions in red colour.

**Editor Comments:**

**Comment 1:** please re-format your manuscript by following the PLOS author guidlines.

**Response:** Thanks for your advice. We have re-formatted the manuscript by following the PLOS author guidlines.

**Comment 2:** please draw out your conclusion based on your studies' result.

**Response:** Thanks for your suggestion. We have rewritten the conclusion. All conclusions are based on our studies’ result.

**Comment 3:** please highlight the scientific question and your contributions.

**Response:** thanks. We have highlighted the scientific question and contributions in Bold type.

**Journal** **Requirements:**

**Requirement 1:** Please ensure that your manuscript meets PLOS ONE's style requirements, including those for file naming.

**Response:** Thanks for your advice. We have re-formatted the manuscript by following the PLOS author guidlines.

**Requirement 2:** If you wish to make changes to your Data Availability statement, please describe these changes in your cover letter and we will update your Data Availability statement to reflect the information you provide.

**Response:** Thanks for reminding me. We have changed our Data Availability statement in cover letter.

**Requirement 3:** Please amend your list of authors on the manuscript to ensure that each author is linked to an affiliation.

**Response:** Thanks for reminding me. We have amened the list of authors and the affiliations.

**Requirement 4:** Please upload a new copy of Figure 3 as the detail is not clear.

**Response:** We have uploaded a new copy of Figure 3. We utilized the PACE tool to ensure that figures meet PLOS requirements.

**Requirement 5:** Please ensure that you refer to Figure 3 and 4 in your text as, if accepted, production will need this reference to link the reader to the figure.

**Response:** Thanks for your advice. We have referred to Figure 3 and 4 in our text.

**Requirement 6:** We note you have included a table to which you do not refer in the text of your manuscript. Please ensure that you refer to Table 1 in your text; if accepted, production will need this reference to link the reader to the Table.

**Response:** Thanks for your advice. We have referred to Table 1 in our text.

**Requirement 7:** Please include captions for your Supporting Information files at the end of your manuscript, and update any in-text citations to match accordingly.

**Response**: Thanks. We have provided the captions of supporting information files at the end of the manuscript, and updated the in-text citations to match accordingly.

We tried our best to improve the manuscript and we appreciate for Editors’ warm work earnestly, and hope that the correction will meet with approval.

Once again, thank you very much for your comments and suggestions.

Your sincerely

Yi Ding

270652247@qq.com
