# Supplementary material for: The impact of agricultural industrial agglomeration on farmers’ income: An influence mechanism test based on a spatial panel model
Source: PLoS One. 2023 Sep 8;18(9):e0291188. doi: 10.1371/journal.pone.0291188 (PMC10491000; doi:10.1371/journal.pone.0291188)
Supplement: Attachment — Submitted filename: Response to Reviewers.doc [file pone.0291188.s007.doc]

Response to reviewers

Dear editor and reviewers,

Thank you for offering us an opportunity to improve the quality of our submitted manuscript(”The impact of agricultural industrial agglomeration on farmers' income: an influence mechanism test based on a spatial panel model ”). We appreciated very much the reviewers’ constructive comments. In this revision, we have addressed all of these comments. We hope the revised manuscript has now met the publication standard of our journal.

We highlighted all the revisions in red colour.

**Journal** **Requirements:**

**Requirement 1:** Please review your reference list to ensure that it is complete and correct. If you have cited papers that have been retracted, please include the rationale for doing so in the manuscript text, or remove these references and replace them with relevant current references. Any changes to the reference list should be mentioned in the rebuttal letter that accompanies your revised manuscript. If you need to cite a retracted article, indicate the article’s retracted status in the References list and also include a citation and full reference for the retraction notice.

**Response:** Thanks for your advice. We checked the reference list and no retracted papers were cited. Our reference list has not changed.

**Requirement 2:** Please upload a new copy of Figure 1,2,3 and 4 as the detail is not clear.

**Response:** Thanks for your advice. We have uploaded all the figures again. We set the picture parameters according to the requirements of our journal, and uploaded to PACE for modification. We hope the figures has now met the publication standard.

**Requirement 3:** Please remove your figures from within your manuscript file, leaving only the individual TIFF/EPS image files.  These will be automatically included in the reviewer’s PDF.

**Response:** Thanks. Figures were removed from the manuscript, leaving only the individual TIFF image files.

**Reviewers' comments:**

**comment 1:** Clarify how the research connects with the SDG goals: The paper should clearly explain how the research relates to the SDG goals, specifically SDG 1 (no poverty) and SDG 2 (zero hunger). The authors should make a connection between the study's findings and how it contributes to these SDGs.

Highlight the role of agricultural industrial agglomeration in promoting sustainable development: The paper could highlight the role of agricultural industrial agglomeration in promoting sustainable development, specifically in the context of SDG 8 (decent work and economic growth) and SDG 9 (industry, innovation and infrastructure). The authors could emphasize how agglomeration can contribute to improving productivity and creating decent work opportunities.

Discuss the implications of the findings on policy and practice: The paper should discuss the implications of the findings on policy and practice, specifically in the context of SDG 17 (partnerships for the goals). The authors could emphasize how the findings can inform policy decisions related to promoting agricultural industrial agglomeration and improving farmers' income.

**Response:** Thanks for your advice. We have rewritten part of the introduce to clarify the importance of agro-industrial agglomeration to the Sustainable Development Goals. In addition, we have added a section "4.5discussion" to discuss the contribution of research findings to the Sustainable Development Goals (SDG1,SDG2,SDG8,SDG9,SDG17). Finally, we have proposed some useful suggestions for the policy and practice of sustainable development based on our findings.

**comment 2:** Consider the potential negative impacts of agricultural industrial agglomeration: The paper could also consider the potential negative impacts of agricultural industrial agglomeration on the environment and local communities, in the context of SDG 13 (climate action) and SDG 15 (life on land). The authors could discuss how agglomeration can lead to increased environmental degradation and social conflicts, and how these negative impacts can be mitigated.

**Response:** Thanks for your advice. In Section 4.5, we discussed the possible negative impacts of agro-industrial agglomeration, such as environmental degradation(SDG13) and community conflicts(SDG15), and proposed solutions.

**comment 3:** send the data with STATA do file to verify the results.

**Response:** Thanks for your advice. We have submitted the raw data and STATA do files in the attachment. In order to provide accurate data, we ran all the tests again. We added time and individual effect test, as well as operations such as tail reduction. Therefore, some results were slightly changed, but the overall conclusion was unchanged. Changes in the regression results are marked in red.

We tried our best to improve the manuscript and we appreciate for Editors’ warm work earnestly, and hope that the correction will meet with approval.

Once again, thank you very much for your comments and suggestions.

Your sincerely

Yi Ding

270652247@qq.com
